# Supplementary material for: Verification of the effect of data-driven brain motion correction on PET imaging
Source: PLoS One. 2024 Jul 5;19(7):e0301919. doi: 10.1371/journal.pone.0301919 (PMC11226115; doi:10.1371/journal.pone.0301919)
Supplement: Attachment — Submitted filename: Response to Reviewers R3.docx [file pone.0301919.s001.docx]

Manuscript PONE-D-24-01135

Response to Reviewers

Dear Dr. Khan Bahadar Khan

Thank you for giving us the opportunity to submit a revised draft of the manuscript “Verification of the effect of Data-Driven Brain Motion Correction on PET imaging ” for publication in the PLOS ONE. We appreciate the time and effort that you and the reviewers dedicated to providing feedback on our manuscript and are grateful for the insightful comments on and valuable improvements to our paper.

We have incorporated most of the suggestions made by the reviewers. Those changes are highlighted within the manuscript. Please see below, in blue, for a point-by-point response to the reviewers’comments and concerns. All page numbers refer to the revised manuscript file with tracked changes.

**Reviewers' Comments to the Authors:**

**Dear Reviewer ＃１**

Thank you very much for reviewing our manuscript and offering valuable advice. We have addressed your comments with point-by-point responses and revised the manuscript accordingly.

1. Abstract. A big limitation of the study is that the method was only validated with Hoffman phantom but not human brain. This should be stated explicitly.

Author response: We have made some modifications to the introduction and conclusion parts of the abstract as follows.

Introduction: *In this study, we investigated the effectiveness of data-driven brain motion correction techniques on positron emission tomography/computed tomography images using a Hoffman phantom, involving continuous rotational motion and tilting motion, each expanded up to approximately 20 degrees. (* *page 2, lines 41-43)*

Conclusions: *However, a significant limitation of this study is the exclusive validation of the proposed method using a Hoffman phantom; its applicability to the human brain has not been investigated. Further research involving human subjects is necessary to assess the generalizability and reliability of the presented motion correction technique in real clinical scenarios. (Page 3, lines 63-67)*

1. Abstract. “% contrast and normalized mean squared error were improved after correction.” Numbers would talk more than simply stating “improved”.

Author response: We have included NMSE values and additional analyzed SSIM values in the results section of the abstract.

*Normalized Mean Squared Error (NMSE) results demonstrated the effectiveness of DDBMC in compensating for rotational and tilting motions during PET imaging. In Cases 1 and 2 involving rotational motion, NMSE decreased from 0.15-0.2 to approximately 0.01 with DDBMC, indicating a substantial reduction in differences from the reference image across various brain regions. In the Structural Similarity Index (SSIM), DDBMC improved it to above 0.96 Contrast assessment revealed notable improvements with DDBMC. In continuous rotational motion, % contrast increased from 42.4% to 73.5%, In tilting motion, % contrast increased from 52.3% to 64.5%, eliminating significant differences from the static reference image. These findings underscore the efficacy of DDBMC in enhancing image contrast and minimizing motion induced variations across different motion scenarios. (Page 2, lines 50 - Page 3, lines 60)*

1. Abstract. “Although the effectiveness of brain motion correction was confirmed in this experiment, it is necessary to understand the relationship between the range of motion and limitations in brain motion correction processing because there might be more complex movements in clinical practice.” I consider you are not saying results. Please move to conclusions.

Author response: We have revised the concluding sentence in light of the significant content of your proposal.

*DDBMC processing can effectively compensate for continuous rotational and tilting motion of the head during PET, with motion angles of approximately 20 degrees. However, a significant limitation of this study is the exclusive validation of the proposed method using a Hoffman phantom; its applicability to the human brain has not been investigated. Further research involving human subjects is necessary to assess the generalizability and reliability of the presented motion correction technique in real clinical scenarios. (Page 3, lines 61-67)*

1. Introduction. “tau PET imaging preparations are also being investigated.” What is “imaging preparations”? Please define.

Author response: *Tau PET tracers such as F-18-PM-PBB3 and F-18-SNFT-1 are being studied domestically and internationally, with anticipated clinical applications in the future [6, 7]. (Page 3, lines 76-78)*

*［6］Endo H, Tagai K, Ono M, Ikoma Y, Oyama A, Matsuoka K, et al. A Machine Learning-Based Approach to Discrimination of Tauopathies Using [(18) F]PM-PBB3 PET Images. Mov Disord. 2022;37(11):2236-46. Epub 20220828. doi: 10.1002/mds.29173. PubMed PMID: 36054492; PubMed Central PMCID: PMCPMC9805085.*

*［7］Harada R, Lerdsirisuk P, Shimizu Y, Yokoyama Y, Du Y, Kudo K, et al. Preclinical Characterization of the Tau PET Tracer [(18)F]SNFT-1: Comparison of Tau PET Tracers. J Nucl Med. 2023;64(9):1495-501. Epub 20230615. doi: 10.2967/jnumed.123.265593. PubMed PMID: 37321821.*

1. Introduction. “Movement during the examination might result in inaccurate images.” An image cannot be inaccurate, only its quantification can.

Author response: We have added and revised the citation references.

*The most cited effects of patient motion are frame image misalignment, which affects the dynamic analysis of dynamic protocols, and resolution loss due to motion blurring in the frame. Moreover, motion during scans can cause mis-estimation in tracer kinetic modeling, introducing inaccuracies in the quantitative analysis of the data [15]. (Page 4, lines 85-89)*

*［15］Anton-Rodriguez JM, Sibomana M, Walker MD, Huisman MC, Matthews JC, Feldmann M, et al., editors. Investigation of motion induced errors in scatter correction for the HRRT brain scanner2010: IEEE.*

1. Introduction. “Research and development of technologies to compensate for head motion is underway, including hardware-based motion tracking methods that track markers attached to the head with an external device…” Hardware based motion measure and compensation methods have been mature for more than a decade. So it is not appropriate to say they are still underway.

Author response: We have added and revised the citation references.

*Several techniques have been implemented to compensate for head motion, including hardware-based motion tracking methods that utilize an external device to track a marker attached to the head [15, 16], and marker-less tracking methods that employ a charged-coupled device camera to extract the surface shape of the object [17, 18]. (Page 4, lines 89-93)*

*［17］Zeng T, Lu Y, Jiang W, Zheng J, Zhang J, Gravel P, et al. Markerless head motion tracking and event-by-event correction in brain PET. Phys Med Biol. 2023;68(24). Epub 20231212. doi: 10.1088/1361-6560/ad0e37. PubMed PMID: 37983915; PubMed Central PMCID: PMCPMC10713921.*

*［18］Iwao Y, Akamatsu G, Tashima H, Takahashi M, Yamaya T. Brain PET motion correction using 3D face-shape model: the first clinical study. Ann Nucl Med. 2022;36(10):904-12. Epub 20220719. doi: 10.1007/s12149-022-01774-0. PubMed PMID: 35854178; PubMed Central PMCID: PMCPMC9515015*

1. Introduction. “charged-coupled device(CCD)” No need to state the abbreviation if it is only mentioned once.

Author response: The abbreviation has been removed.

*charged-coupled device camera (Page 4, lines 92)*

1. Introduction. “A data-driven brain motion correction (DDBMC) technique…” Although reader can find implementation details themselves, it is still helpful to introduce how it works briefly.

Author response: We have added and revised the citation references.

*In DDBMC, image reconstruction is performed with ultra-short frames (≤1 second) from listmode data . Motion is estimated by performing image rigid registration between each frame and a selected reference frame, allowing for motion-corrected listmode reconstruction. Each frame is reconstructed using maximum-likelihood expectation maximization without subsets, because of the low number of events in each frame. Since accurate quantification is not crucial, scatter correction and point spread function modeling are omitted, and only a few iterations are carried out. If necessary, you can use the standard attenuation correction map generated from CT images[5, 23]. (Page 4, lines97- Page 5. lines104)*

*［23］Revilla EM, Gallezot JD, Naganawa M, Toyonaga T, Fontaine K, Mulnix T, et al. Adaptive data-driven motion detection and optimized correction for brain PET. Neuroimage. 2022;252:119031. Epub 20220304. doi: 10.1016/j.neuroimage.2022.119031. PubMed PMID: 35257856; PubMed Central PMCID: PMCPMC9206767.*

1. Introduction. “In the present study, we validated our method…” Consider change “our” to “the” as it is not proposed by the authors.

Author response: *we validated the method using PET/CT (Page 5, lines 105)*

1. Materials and methods. GE HealthCare -> GE Healthcare

Author response: Since the division in January 2023, The company name has been changed to GE HealthCare. [Link: <https://www.gehealthcare.com/>]

We will use the notation '*GE HealthCare*' this time. *(Page 5, lines 113)*

1. Lutetium based scintillators (LBSs). Do you mean LSO or something else?

Author response: The following correction is made.

*It consists of a 128-slice CT system and a 4-ring PET system with lutetium-yttrium oxyorthosilicate (LYSO) crystals and silicon photomultiplier-based (SiPM) detectors providing a 20-cm axial field of view and a 70-cm transaxial field of view. (Page 5, lines 114-117)*

1. “The LBSs and SiPMs detectors enable time resolution below 380 ps and time-of-flight compatibility.” Consider rephrasing it to “The LBSs and SiPMs detectors enable time-of-flight compatibility with time resolution below 380 ps.” I did not go through the whole paper, but it is likely other sentences need to be rephrased too.

Author response: We will correct as you indicated.

*The LYSO and SiPM detectors enable time-of-flight compatibility with time resolution below 380 ps. [24, 25]. (Page 55, lines 119-120)*

1. “Although the range of motion is considered sufficient, it is assumed that a variety of movements will be mixed in actual.” Although you simulated three cases of movement, they still cannot represent the real case. For example, a real head movement can be the combination of six DOFs. Can you comment on this?

Author response: We have added this as a limitation to this study.

*In clinical examinations, head motion involves a combination of six degrees of freedom (6-DOF). Spangler-Bickell et al. reported the effectiveness of DDBMC even in clinical cases [20]. This suggests that DDBMC is useful for various movements. However, understanding the limits of the correction region for movements based on the combination of 6-DOF is crucial, and further verification is deemed necessary in the future. (Page 18, lines 397-402)*

1. Line 133, 1 second/frame was used. Have you performed denoising? “list data” should be “listmode data”.

Author response: The reconstruction method for 1-second data does not include noise removal.

*For reconstruction of the ultra-short frames, the maximum likelihood expectation maximization method was used. Reconstructions were performed with six iterations, with no attenuation or scatter corrections [5]. (Page 7, lines 172- Page 8, lines 174)*

We have made changes to all instances where “list data”was mentioned and replaced it with “listmode data.” *(page 4, line 100, page 7, line 170, page 10, line 225,227, 234, 238, page 18, line 387)*

1. Contrast and NMSE are good metrics to evaluate the correction. It would be nice to see SSIM to reflect the recovery of global features.

Author response: As you suggested, we have incorporated SSIM analysis as an image evaluation method and made the following modifications.

Method: *Furthermore, a structure similarity index (SSIM) was used to measure the degree of similarity between the reference image and an image of interest [28]. The SSIM is sensitive to contrast, luminance, and structures within an image, with values ranging from 0 to 1. As SSIM approaches 1, the similarity with the reference image increases. Of the total of Seventy-one slices from one-bed scan imaging, it evaluated 43 slices delineated from the parietal lobe to the cerebellum. SSIM calculations were performed using skimage 0.21.0 and Python 3.8.10. (Page 8, lines 194- Page 9, lines 200)*

Result: ***Normalized mean squared error and structure similarity index***

*The results of NMSE for each slice, comparing images with rotational or tilting motion to the reference images taken in a static state, are presented in Fig 7. Additionally, Table 2. provides the mean and standard deviation values of NMSE and SSIM for all slices. In the case of images with rotational motion (Cases 1 and 2), NMSE ranged from 0.15 to 0.2 from the basal ganglia to the cerebellum, indicating a significant difference from the reference images. With DDBMC, the variation based on slice position and reduces differences from the reference images. With DDBMC, mean NMSE decreases to approximately 0.01 for both Cases 1 and 2. In terms of SSIM, there is a significant improvement, with Case 1 increasing from 0.892 to 0.968, and Case 2 increasing from 0.860 to 0.967. In Case 3, involving tilting motion, the differences in NMSE values in the basal ganglia were smaller than those in rotational motion. However, the NMSE values in the vertex and cerebellar regions were larger. With DDBMC, NMSE significantly improved from 0.262 to 0.016, and SSIM increased from 0.874 to 0.962. (Page 13, lines 289- 301)*

Discussion: *Furthermore, the SSIM with DDBMC showed a significantly higher value of 0.96 or higher than that without DDBMC. We believe that this result is consistent with the fact that all cases after DDBMC as "Diagnostic" in the visual evaluation by the specialist physicians. (Page 17, lines 371-374)*

1. “The results of the visual evaluation are shown in Table 1” Four average scores are 4, which suggests all raters have consensus of scoring 4? Without is out of boundary.

Author response: The text has been revised due to unclear content, and furthermore, Table 1 has been added.

*In images without DDBMC, the average scores were 0.2 for Case 1 and 1.0 for Case 2, indicating “Not diagnostic”. For Case 3, the average score was 2.6. All evaluators judged the reference images and images with DDBMC as “Diagnostic”. (Page 12, lines 278-281)*

1. “The closer NMSE is to 0, the closer the image with motion is to the reference image” Consider remove this sentence, as it already appeared in Methods.

Author response: We have removed the part you indicated.

1. Discussion. “However, it is difficult for the subject to remain still because of the long time needed to acquire images”. New scanners can scan head within a minute. The associated challenge will then be the vulnerability of ultra-high resolution.

Author response: We have made the following corrections.

*Because head movements during brain PET imaging can lead to image degradation, the head is immobilized during imaging. However, due to the extended time required for image acquisition, it can be challenging for all subjects to remain completely still, depending on their age and physical condition. In short-duration imaging, there is a potential for decreased resolution. Therefore, techniques are needed to minimize the effect of head motion on the images. (Page 15, lines 327- Page 16, 332)*

1. “In this study, we used DDBMC, which was devised as a correction method that does not require an external device.” Some important references are missing. There are other data-driven method papers in recent years. For example:Rezaei et al. PMB 2021, Rigid motion tracking using moments of inertia in TOF-PET brain studies. Sun T. et al. PMB 2022, An iterative image-based inter-frame motion compensation method for dynamic brain PET imaging.

Author response: As suggested, we have added the article to the Reference.

*A data-driven brain motion correction (DDBMC) technique has been developed [19-22]. (Page 4, lines 96-97)*

*[21]Rezaei A, Spangler-Bickell M, Schramm G, Van Laere K, Nuyts J, Defrise M. Rigid motion tracking using moments of inertia in TOF-PET brain studies. Phys Med Biol. 2021;66(18). Epub 20210913. doi: 10.1088/1361-6560/ac2268. PubMed PMID: 34464941.*

*[22]Sun T, Wu Y, Bai Y, Wang Z, Shen C, Wang W, et al. An iterative image-based inter-frame motion compensation method for dynamic brain PET imaging. Phys Med Biol. 2022;67(3). Epub 20220202. doi: 10.1088/1361-6560/ac4a8f. PubMed PMID: 35021156.*

1. A limitation is the study was only validated in static scan but not dynamic scan.

Author response: Added as a limitation to this study.

*The results of this study mainly apply to static data, where changes in tracer distribution over time are minimal. In dynamic data, which captures changes in tracer distribution, it is necessary to update the reference image regularly. The optimal strategy for handling this is required. (Page 19, lines 410-413)*

**Dear Reviewer ＃２**

Thank you very much for reviewing our manuscript and offering valuable advice. We have addressed your comments with point-by-point responses, and revised the manuscript accordingly.

1. Page 4, line 78. Reference 15 is not related to hardware-based motion tracking. I suggest changing appropriate reference here.

Author response: We have removed the cited references you pointed out and added the following references.

*Several techniques have been implemented to compensate for head motion, including hardware-based motion tracking methods that utilize an external device to track a marker attached to the head [15, 16], and marker-less tracking methods that employ a charged-coupled device camera to extract the surface shape of the object [17, 18]. (Page 4, lines 89 -90)*

1. In the Introduction section, the author did not introduce the data-driven method, and there is no review for other data-driven head motion correction methods. Such as centroid of distribution.

Author response: We have added and revised the citation references.

*In DDBMC, image reconstruction is performed with ultra-short frames (≤1 second) from listmode data. Motion is estimated by performing image rigid registration between each frame and a selected reference frame, allowing for motion-corrected list-mode reconstruction. Each frame is reconstructed using maximum-likelihood expectation maximization without subsets, because of the low number of events in each frame. Since accurate quantification is not crucial, scatter correction and point spread function modeling are omitted, and only a few iterations are carried out. If necessary, you can use the standard attenuation correction map generated from CT images[5, 23]. (Page 5, lines 97-104)*

*［23］Revilla EM, Gallezot JD, Naganawa M, Toyonaga T, Fontaine K, Mulnix T, et al. Adaptive data-driven motion detection and optimized correction for brain PET. Neuroimage. 2022;252:119031. Epub 20220304. doi: 10.1016/j.neuroimage.2022.119031. PubMed PMID: 35257856; PubMed Central PMCID: PMCPMC9206767.*

1. Figures are hard to read and there are no captions. Consider adding the captions for the figures, and make the figure self-explanatory. Replace Figure 5 and 6 with vector graphics.

Author response: We added captions to the figures and replaced them with vector graphics.

1. There is no comparison between Vicra motion tracking result and data-driven motion tracking. The author may consider including the quantitative measurements between the distance of the two measurements.

Author response: This study was not intended to be a direct comparison between Polaris and DDBMC. Due to misleading wording, the following corrections have been made.

Results: *Fig 4 shows the motion plots used in the ultra-short (1 second) DDBMC for Polaris Vicra optical tracking and listmode data in each case. The top row shows motion plots obtained from Polaris Vicra external devices. The bottom row is a motion plot of displacements computed from ultrashort frame images of the listmode data . Fig 4 (A) shows the relationship between displacement in the X, Y, and Z directions and elapsed time, and Fig 4 (B) shows the relationship between angular displacement in the X, Y, and Z directions and elapsed time. Comparison of motion corrected images from data of both methods resulted in the mean values (standard deviation) of NMSE: 0.005 (0.001) for Case 1, 0.008 (0.002) for Case 2, and 0.011 (0.003) for Case 3. We confirmed that displacement calculations comparable to Polaris Vicra optical tracking are possible even from ultrashort (1 second) images of listmode data (Page 10, lines 224- 234)*

1. Page 10, line 235. Reference 17 and 18 are old references, the author should consider reporting the latest research for the markerless motion tracking.

Author response: As you indicated, we have changed the text and added references.

*Slipsager JM et al. demonstrated the utility of a motion tracking system based on computer vision technology, employing a structured optical surface scanner. The system utilizes a synchronized optical modulator and camera for continuous scanning of the patient's face. In contrast to marker-based solutions, this approach eliminates the need for attaching optical markers, resulting in a reduction in clinical preparation time [29]. (Page 16, lines 334- 339)*

*[29]* *Slipsager JM, Ellegaard AH, Glimberg SL, Paulsen RR, Tisdall MD, Wighton P, et al. Markerless motion tracking and correction for PET, MRI, and simultaneous PET/MRI. PLoS One. 2019;14(4):e0215524. Epub 20190419. doi: 10.1371/journal.pone.0215524. PubMed PMID: 31002725; PubMed Central PMCID: PMCPMC6474595.*

1. In the discussion, the author may want to discuss the recent deep learning data-driven methods for brain PET head motion correction.

Author response: Thank you very much for your excellent suggestion.

*Additionally, Zeng T et al. introduced a novel structure for deep neural networks for head motion estimation using supervised learning [17, 31]. Tumpa TR et al. also proposed an unsupervised deep learning approach for brain motion correction through 3D image registration [32]. While keeping an eye on these new technologies, I aim to promote the utilization of DDBMC in clinical examinations. (Page 19, lines 416-420)*

We would like to express our sincere gratitude for your invaluable time, expertise, and thoughtful feedback during the review process. Your constructive comments and insightful suggestions have significantly contributed to enhancing the quality and clarity of our manuscript. We look forward to your continued support in the future.

Hayato Odagiri

E-mail:hayato.odagiri.e2@tohoku.ac.jp
