# Supplementary material for: Verification of the effect of data-driven brain motion correction on PET imaging
Source: PLoS One. 2024 Jul 5;19(7):e0301919. doi: 10.1371/journal.pone.0301919 (PMC11226115; doi:10.1371/journal.pone.0301919)
Supplement: Attachment — Submitted filename: Response to Reviewers 2nd.docx [file pone.0301919.s002.docx]

Manuscript PONE-D-24-01135

Response to Reviewers

Dear Dr. Khan Bahadar Khan

Thank you for giving us the opportunity to submit a revised draft of the manuscript “Verification of the effect of Data-Driven Brain Motion Correction on PET imaging ” for publication in the PLOS ONE. We appreciate the time and effort that you and the reviewers dedicated to providing feedback on our manuscript and are grateful for the insightful comments on and valuable improvements to our paper.

We have incorporated most of the suggestions made by the reviewers. Those changes are highlighted within the manuscript. Please see below, in blue, for a point-by-point response to the reviewers’comments and concerns. All page numbers refer to the revised manuscript file with tracked changes.

**Reviewers' Comments to the Authors:**

**Dear Reviewer ＃１**

Thank you very much for reviewing our manuscript and offering valuable advice. 　We have addressed your comments with point-by-point responses and revised the manuscript accordingly.

1. Thank you for addressing most of my comments. My only remaining concern is for comment no. 14. Do you have an example image? And for a patient scan, the image noise for 1-sec frame could be larger than you ideal phantom image.

Author response: Thank you for your suggestion. We have added a reference [20] showing 1-second images in clinical data.

*DDBMC makes ultra-short (approximately 1 second/frame, depending on the data) reconstructions from listmode data and estimates motion between these ultra-short images and a reference image at the beginning of acquisition (30 seconds/frame) using rigid registration [20].* *(* *page 7, lines 173)*

**Dear Reviewer ＃2**

Thank you very much for reviewing our manuscript and offering valuable advice. 　We have addressed your comments with point-by-point responses and revised the manuscript accordingly.

1. The hardware-based motion tracking requires a tracking device and additional setup, but it does not require a device to be attached to the patient. Usually, the device was fixed on the PET scanner gantry. The author should correct this sentence.

Author response: Due to the confusing wording, we have revised it as follows.

*The motion tracking method using Polaris Vicra requires the attachment of light-reflective markers to the patient, which is time-consuming to set up and may cause the attached markers to come off due to movement [19]. (* *page 4, lines 93-96)*

*[19]. Woo S-K, Watabe H, Choi Y, Kim KM, Park CC, Bloomfield PM, et al. Sinogram-based motion correction of pet images using optical motion tracking system and list-mode data acquisition. IEEE Transactions on Nuclear Science. 2004;51(3):782-8.*

2. references 24 and 25 are related to the reconstruction methodology. The author should consider removing them and adding the system performance paper of the Discovery PET for reference.

Author response: We have added references "24, and 25" regarding the performance of the PET scanner.　 ( page 5, lines 121)

*[24].Hsu DFC, Ilan E, Peterson WT, Uribe J, Lubberink M, Levin CS. Studies of a Next-Generation Silicon-Photomultiplier-Based Time-of-Flight PET/CT System. J Nucl Med. 2017;58(9):1511-8. Epub 20170427. doi: 10.2967/jnumed.117.189514. PubMed PMID: 28450566.*

*[25]. Chicheportiche A, Marciano R, Orevi M. Comparison of NEMA characterizations for Discovery MI and Discovery MI-DR TOF PET/CT systems at different sites and with other commercial PET/CT systems. EJNMMI Phys. 2020;7(1):4. Epub 20200114. doi: 10.1186/s40658-020-0271-x. PubMed PMID: 31938953; PubMed Central PMCID: PMCPMC6960280.*

3. reference 22 is not related to the DDBMC. The author should consider correcting this error. In addition, the other data-driven MC methods should be introduced, like COD (reference 23) and method in reference 22.

Author response: Reference 22 has been removed. ( page 4, lines 98)

The following sentence was added to the discussion.

*Revilla et al. proposed a data-driven method, the three-dimensional center of distribution, reporting significant reduction in blurring due to head motion [23]. It has been noted that this method may not be able to accommodate large changes in tracer activity within the brain. Verification is needed in the present study regarding the DDBMC examined here in cases of significant changes in tracer activity within the brain. (* *page 19, lines 414-419)*

4. reference 17 is a device-based motion correction method. Here the author wants to discuss deep learning method for PET head motion correction, I would recommend reference 31. The other possible reference related to the topic:

Reimers, Erik, Ju-Chieh Kevin Cheng, and Vesna Sossi. "Deep Learning Aided Intra-Frame Motion Correction for Low-Count Dynamic Brain PET." IEEE Transactions on Radiation and Plasma Medical Sciences (2023).

Zeng, Tianyi, et al. "Supervised deep learning for head motion correction in PET." International Conference on Medical Image Computing and Computer-Assisted Intervention. Cham: Springer Nature Switzerland, 2022.

Author response: Reference 17 has been removed, and we have added reference "34" as a relevant citation. ( page 19, lines 423)

*[34]. Zeng T, Zhang J, Revilla E, Lieffrig EV, Fang X, Lu Y, et al. Supervised Deep Learning for Head Motion Correction in PET. Med Image Comput Comput Assist Interv. 2022;13434:194-203. Epub 20220916. doi: 10.1007/978-3-031-16440-8_19. PubMed PMID: 38107622; PubMed Central PMCID: PMCPMC10725740.*

We would like to express our sincere gratitude for your invaluable time, expertise, and thoughtful feedback during the review process. We look forward to your continued support in the future.

Hayato Odagiri

E-mail:hayato.odagiri.e2@tohoku.ac.jp
